# Pharmacovigilance analysis of immune checkpoint inhibitor-associated optic nerve toxicity based on the FAERS database

**DOI:** 10.3389/fphar.2026.1845963

**Published:** 2026-08-24

**Authors:** Hejing Bao, Liping Lin, Wanmin Huan, Hehong Bao, Haoran Su, Xi Luo, Caijiu Deng, Jiazhu Hu, Xiaolong Cao, Xiaosong Wu

**Affiliations:** 1 Department of Oncology, The Affiliated Panyu Center Hospital, Guangzhou Medical University, Guangzhou, Guangdong, China; 2 Cancer Institute of Panyu District, The Affiliated Panyu Center Hospital, Guangzhou Medical University, Guangzhou, Guangdong, China; 3 Department of Psychosomatic Medicine, Chongqing University Three Gorges Hospital, Chongqing, China; 4 Department of Ophthalmology, The First People’s Hospital of Foshan (Foshan Hospital Affiliated to Southern University of Science and Technology), School of Medicine, Southern University of Science and Technology, Foshan, Guangdong, China

**Keywords:** disproportionality analysis, FAERS, immune checkpoint inhibitors, optic nerve toxicity, pharmacovigilance

## Abstract

**Background:**

Immune checkpoint inhibitors (ICIs) can cause rare but potentially vision-threatening optic nerve toxicity. This study aimed to characterize ICI-associated optic nerve toxicity using the U.S. Food and Drug Administration Adverse Event Reporting System (FAERS).

**Methods:**

FAERS reports from the first quarter of 2004 to the second quarter of 2025 were screened for optic nerve toxicity associated with five ICIs: pembrolizumab, nivolumab, atezolizumab, durvalumab, and ipilimumab. Baseline characteristics, temporal trends, disproportionality signals, time-to-onset patterns, and potential associated factors were analyzed.

**Results:**

A total of 53,522 pembrolizumab-related, 67,178 nivolumab-related, 23,394 atezolizumab-related, 13,246 durvalumab-related, and 18,488 ipilimumab-related reports were included. Optic nerve toxicity was reported mainly in middle-aged and older adults, with the United States contributing most reports. In recent years, the number of relevant reports showed an increasing trend. Disproportionality analysis detected significant signals for optic nerve toxicity and related optic nerve events. Time-to-onset analysis revealed that all five ICIs exhibited a “wear-out failure” pattern, suggesting that the reporting probability of optic nerve-related adverse events might increase gradually with prolonged exposure. Age was the most important feature, followed by sex. Among concomitant medications, gabapentin showed a relatively strong association; among comorbidities, erosive gastritis and hypophysitis were more commonly reported.

**Conclusion:**

This FAERS-based pharmacovigilance study identified optic nerve toxicity as a clinically relevant adverse event associated with five ICIs. Age and sex were key associated factors. These findings support closer ophthalmic monitoring during ICI therapy.

## Introduction

Optic nerve toxicity is an uncommon but potentially vision-threatening adverse event that can cause acute or subacute visual loss (Song et al., 2022). Clinically, optic nerve disorders are broadly classified as typical or atypical. Typical cases often follow a recognizable demyelinating pattern and may occur in isolation or in association with multiple sclerosis, whereas atypical cases may be related to neuromyelitis optica spectrum disorder, infection, or autoimmune disease (Žorić and Čolak, 2025; Phuljhele et al., 2021). Because optic nerve toxicity is frequently misdiagnosed, delayed recognition may compromise visual recovery (De Lott et al., 2022). Therefore, identifying drug-related optic nerve toxicity is important for early monitoring, risk assessment, and mechanistic investigation.

Immune checkpoint inhibitors (ICIs) are widely used in oncology and hematology (Pedrero Prieto et al., 2024). Although these agents enhance antitumor immunity, they can also disrupt immune tolerance and trigger immune-related adverse events (Fletcher and Johnson, 2024; Sun et al., 2023). Neurological toxicities are relatively rare, but when they occur, they may be severe and irreversible; optic nerve toxicity is among the most clinically important ocular complications (Bomze et al., 2022; Ahluwalia and Kohli, 2021). ICI-related optic nerve injury may present as papillitis or optic neuritis and may occur in the setting of broader immune-mediated neurotoxicity with poor visual outcomes (Cuzzubbo et al., 2026).

Despite increasing clinical recognition, systematic pharmacovigilance data on ICI-associated optic nerve toxicity remain limited. In this study, we used the U.S. Food and Drug Administration Adverse Event Reporting System (U.S. FAERS) to analyze reports of optic nerve toxicity associated with five ICIs—pembrolizumab, nivolumab, atezolizumab, durvalumab, and ipilimumab—from the first quarter of 2004 to the second quarter of 2025. We summarized report characteristics, evaluated temporal trends, assessed time-to-onset patterns, performed disproportionality analyses, and explored potential associated factors. This study provides real-world evidence to improve understanding of the safety profile of ICIs and support clinical monitoring of optic nerve toxicity.

## Materials and methods

### Data source and study design

This study used publicly available data from the U.S. FAERS. Quarterly FAERS ASCII files from the first quarter of 2004 to the second quarter of 2025 were downloaded and analyzed. FAERS is a spontaneous reporting database that collects adverse event reports from healthcare professionals, patients, and other reporters. Because the data are de-identified and publicly available, institutional review board approval and informed consent were not required.

### Drug selection and outcome definition

**FIGURE 1:**
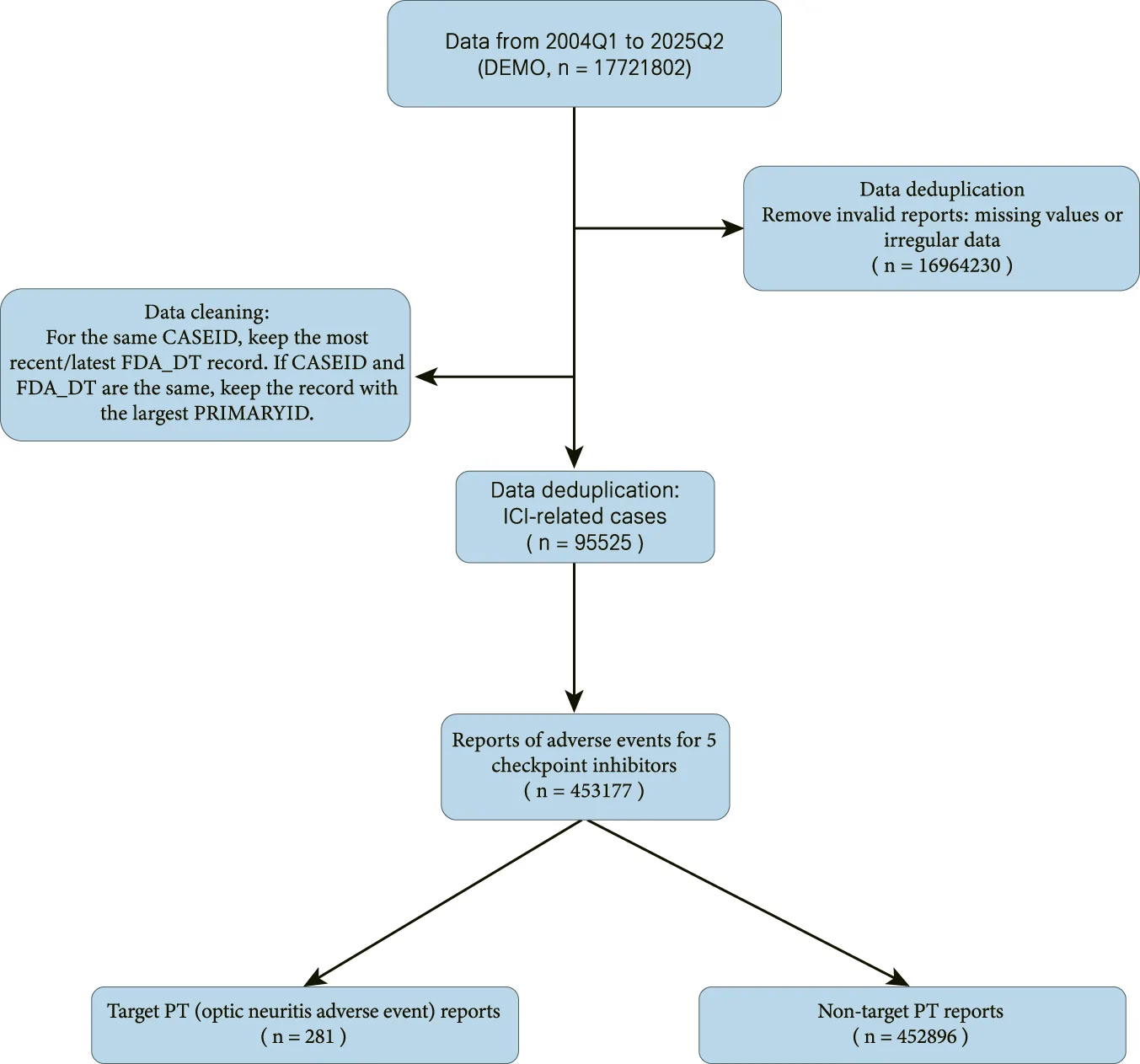
Flow chart showing the analysis process of the study. A detailed description of the selection process of optic nerve toxicity for ICIs in the FAERS.

ICIs were selected as the target drug class, including anti-PD-1 agents (nivolumab, pembrolizumab, cemiplimab), anti-PD-L1 agents (atezolizumab, avelumab, durvalumab), and anti-CTLA-4 agents (ipilimumab, tremelimumab). After data extraction, only five ICIs with optic nerve-related adverse event reports were retained for analysis: pembrolizumab, nivolumab, atezolizumab, durvalumab, and ipilimumab. Other ICIs were excluded because no relevant reports were identified or the number of cases was too small for disproportionality analysis (Figure 1).

Optic nerve-related adverse events were predefined using medical dictionary for regulatory activities (MedDRA) Preferred Terms (PTs), including optic neuritis, optic neuropathy, optic perineuritis, immune-mediated optic neuritis, and neuromyelitis optica spectrum disorder (NMOSD). These terms were grouped together because they represent clinically related inflammatory or demyelinating disorders involving the optic nerve (Figure 1).

### Data cleaning and case selection

According to the FDA-recommended algorithm, FAERS reports were deduplicated using the dplyr package (version 1.1.4) in R software based on CASEID, FDA_DT, and PRIMARYID (Chen et al., 2026), and the report with the latest FDA_DT was retained; if FDA_DT was also identical, the report with the largest PRIMARYID was kept. Invalid reports with missing or inconsistent key information were excluded. Drug information was matched by DRUGNAME to identify target ICIs, and only reports in which the target drug was coded as the primary suspect (PS) were included. Adverse events were coded using MedDRA version 26.1 Preferred Terms and System Organ Classes.

### Disproportionality analysis

A disproportionality analysis was performed to detect signals of optic nerve-related adverse events associated with ICIs. Four signal detection methods were applied: reporting odds ratio (ROR), proportional reporting ratio (PRR), Bayesian confidence propagation neural network (BCPNN), and multi-item gamma Poisson shrinker (MGPS, using empirical Bayesian geometric mean [EBGM]) (Supplementary Table S1). The 2 × 2 contingency table was constructed with a as the number of reports of optic nerve-related adverse events for the drug of interest, b as all other adverse events for the drug of interest, c as optic nerve-related adverse events for all other drugs, and d as all other adverse events for all other drugs (Supplementary Table S2).

A positive signal was defined as meeting the thresholds of all four methods: ROR >1 with the lower bound of the 95% CI > 1; PRR ≥ 2 with χ^2^ ≥ 4 and a ≥ 3; IC025 > 0; and EBGM05 > 2 with a > 0.

### XGBoost modeling and variable processing

To identify clinical features associated with reports of optic nerve toxicity, this study used the XGBoost algorithm to construct a concomitant medication model and a comorbidity model, respectively. The feature pool for the models was determined based on the screening results of prior univariate and multivariate logistic regression analyses. During the data preprocessing stage, records with missing values in PT, AGE, and SEX were removed, and the remaining analysis variables had no missing values in the study dataset. For categorical variables with extremely low frequencies, this study retained their original categories rather than merging or removing them, aiming to preserve potential information in the FAERS database to the greatest extent. Given that XGBoost, as a decision tree-based ensemble learning method, can automatically select features with higher information gain during the node splitting process, the impact of low-frequency categories on the overall model performance was relatively limited.

In the model construction strategy, to prevent data leakage, all optic neuritis-related outcome events were excluded from the predictor variable pool; only clinical information that could be known before the occurrence of adverse events, such as patient demographic characteristics, concomitant medications, and comorbidities, was included in the analysis. The model hyperparameters were configured based on preset values: the maximum tree depth was set to 2 to control model complexity, the learning rate was set to 0.3, the number of iterations was set to 50 to balance fitting capacity and overfitting risk, the objective function was binary logistic regression, and the evaluation metric was log-loss. The complete list of variables included in the final models was provided in Supplementary Table S3.

### Statistical analysis

All analyses were performed using R software (version 4.2.2). Descriptive analyses were conducted using the dplyr package (version 1.1.4), and temporal trend plots were generated using ggplot2 (version 3.5.2). Time to onset was assessed using the survival package (version 3.5.3), and median onset time was summarized by median, interquartile range, and Weibull shape parameter analysis. Logistic regression was performed using the glm function in the stats package (version 4.3.3) to evaluate potential the statistical associations between variables such as age, sex, comorbidities, and concomitant medications and reports of optic nerve toxicity. XGBoost modeling was conducted using the xgboost package (version 1.7.11.1) to assess the relative importance of each feature in the model and to identify clinical variables with high predictive contribution.

## Results

### Baseline characteristics

The baseline characteristics of optic nerve-related adverse event reports associated with ICIs are summarized in Table 1. Overall, female patients were more frequently reported in the atezolizumab and ipilimumab groups, whereas male patients predominated in the pembrolizumab, nivolumab, and durvalumab groups. Most reports involved patients aged 18–85 years, with physicians being the most common reporters. The United States contributed the largest proportion of reports, and hospitalization was the most frequent outcome.

**TABLE 1:** Clinical and reporting characteristics of patients with optic neuritis-related adverse reactions induced by immune checkpoint inhibitors.

Total cases n (%)	Pembrolizumab	Nivolumab	Atezolizumab	Durvalumab	Ipilimumab
	(N = 114)	(N = 102)	(N = 28)	(N = 8)	(N = 34)
Sex					
F	55 (48.2%)	34 (33.3%)	15 (53.6%)	0 (0%)	16 (47.1%)
M	56 (49.1%)	56 (54.9%)	8 (28.6%)	4 (50.0%)	15 (44.1%)
Missing	3 (2.6%)	12 (11.8%)	5 (17.9%)	4 (50.0%)	3 (8.8%)
WT					
Mean (±SD)	70.08 (17.18)	72.87 (21.04)	66.31 (14.39)	NA	77.95 (8.31)
<50 kg	4 (3.5%)	2 (2.0%)	0 (0%)	0 (0%)	0 (0%)
>100 kg	1 (0.9%)	1 (1.0%)	0 (0%)	0 (0%)	0 (0%)
50∼100 kg	26 (22.8%)	23 (22.5%)	10 (35.7%)	0 (0%)	11 (32.4%)
Missing	83 (72.8%)	76 (74.5%)	18 (64.3%)	8 (100%)	23 (67.6%)
Age					
Mean (±SD)	61.11 (18.01)	59.65 (16.23)	57.12 (15.92)	67.03 (9.01)	54.74 (17.64)
<18 years	2 (1.8%)	3 (2.9%)	1 (3.6%)	0 (0%)	1 (2.9%)
18∼64.9 years	41 (36.0%)	41 (40.2%)	14 (50.0%)	2 (25.0%)	19 (55.9%)
65∼85 years	43 (37.7%)	35 (34.3%)	7 (25.0%)	4 (50.0%)	7 (20.6%)
>85 years	3 (2.6%)	0 (0%)	0 (0%)	0 (0%)	1 (2.9%)
Missing	25 (21.9%)	23 (22.5%)	6 (21.4%)	2 (25.0%)	6 (17.6%)
Occp_cod					
Consumer	22 (19.3%)	7 (6.9%)	0 (0%)	0 (0%)	6 (17.6%)
Health professional	17 (14.9%)	30 (29.4%)	7 (25.0%)	0 (0%)	3 (8.8%)
Lawyer	0 (0%)	0 (0%)	0 (0%)	0 (0%)	0 (0%)
Medical doctor	56 (49.1%)	50 (49.0%)	21 (75.0%)	7 (87.5%)	12 (35.3%)
Missing	1 (0.9%)	0 (0%)	0 (0%)	0 (0%)	0 (0%)
Other health-professional	6 (5.3%)	8 (7.8%)	0 (0%)	1 (12.5%)	12 (35.3%)
Pharmacist	12 (10.5%)	7 (6.9%)	0 (0%)	0 (0%)	1 (2.9%)
Registered nurse	0 (0%)	0 (0%)	0 (0%)	0 (0%)	0 (0%)
Reporter_country					
AR	1 (0.9%)	2 (2.0%)	0 (0%)	0 (0%)	0 (0%)
AT	1 (0.9%)	1 (1.0%)	0 (0%)	0 (0%)	0 (0%)
AU	2 (1.8%)	3 (2.9%)	1 (3.6%)	0 (0%)	2 (5.9%)
BE	1 (0.9%)	5 (4.9%)	1 (3.6%)	1 (12.5%)	0 (0%)
BR	2 (1.8%)	0 (0%)	0 (0%)	0 (0%)	0 (0%)
CA	2 (1.8%)	0 (0%)	0 (0%)	0 (0%)	0 (0%)
CH	2 (1.8%)	1 (1.0%)	0 (0%)	0 (0%)	0 (0%)
CN	2 (1.8%)	0 (0%)	1 (3.6%)	0 (0%)	0 (0%)
CO	0 (0%)	1 (1.0%)	0 (0%)	0 (0%)	0 (0%)
Country not specified	0 (0%)	0 (0%)	0 (0%)	0 (0%)	1 (2.9%)
CZ	0 (0%)	1 (1.0%)	0 (0%)	0 (0%)	0 (0%)
DE	5 (4.4%)	7 (6.9%)	1 (3.6%)	0 (0%)	1 (2.9%)
DK	1 (0.9%)	0 (0%)	0 (0%)	0 (0%)	0 (0%)
ES	3 (2.6%)	6 (5.9%)	4 (14.3%)	0 (0%)	0 (0%)
FI	0 (0%)	1 (1.0%)	1 (3.6%)	0 (0%)	0 (0%)
FR	16 (14.0%)	10 (9.8%)	0 (0%)	1 (12.5%)	8 (23.5%)
GB	0 (0%)	1 (1.0%)	1 (3.6%)	1 (12.5%)	0 (0%)
GR	2 (1.8%)	0 (0%)	0 (0%)	0 (0%)	0 (0%)
HU	0 (0%)	1 (1.0%)	0 (0%)	0 (0%)	0 (0%)
I.E.,	0 (0%)	1 (1.0%)	0 (0%)	0 (0%)	0 (0%)
IL	1 (0.9%)	0 (0%)	0 (0%)	0 (0%)	0 (0%)
IT	2 (1.8%)	5 (4.9%)	0 (0%)	0 (0%)	0 (0%)
JP	19 (16.7%)	9 (8.8%)	6 (21.4%)	2 (25.0%)	4 (11.8%)
KR	2 (1.8%)	1 (1.0%)	1 (3.6%)	0 (0%)	0 (0%)
MY	2 (1.8%)	0 (0%)	0 (0%)	0 (0%)	0 (0%)
NL	0 (0%)	0 (0%)	1 (3.6%)	0 (0%)	0 (0%)
NO	1 (0.9%)	0 (0%)	0 (0%)	0 (0%)	0 (0%)
PA	1 (0.9%)	0 (0%)	0 (0%)	0 (0%)	0 (0%)
PE	1 (0.9%)	0 (0%)	0 (0%)	0 (0%)	0 (0%)
PT	0 (0%)	3 (2.9%)	0 (0%)	0 (0%)	0 (0%)
SA	1 (0.9%)	0 (0%)	0 (0%)	0 (0%)	0 (0%)
SE	0 (0%)	1 (1.0%)	0 (0%)	0 (0%)	0 (0%)
SG	0 (0%)	1 (1.0%)	0 (0%)	0 (0%)	0 (0%)
TH	1 (0.9%)	1 (1.0%)	0 (0%)	0 (0%)	0 (0%)
TR	1 (0.9%)	4 (3.9%)	2 (7.1%)	0 (0%)	0 (0%)
UA	0 (0%)	0 (0%)	0 (0%)	1 (12.5%)	0 (0%)
US	42 (36.8%)	36 (35.3%)	8 (28.6%)	2 (25.0%)	17 (50.0%)
United states	0 (0%)	0 (0%)	0 (0%)	0 (0%)	1 (2.9%)
Outcome					
Death	1 (0.9%)	5 (4.9%)	4 (14.3%)	1 (12.5%)	0 (0%)
Disability	11 (9.6%)	6 (5.9%)	2 (7.1%)	2 (25.0%)	1 (2.9%)
Hospitalization	36 (31.6%)	26 (25.5%)	6 (21.4%)	2 (25.0%)	8 (23.5%)
Life-threatening	3 (2.6%)	4 (3.9%)	2 (7.1%)	1 (12.5%)	1 (2.9%)
Other	63 (55.3%)	61 (59.8%)	14 (50.0%)	2 (25.0%)	24 (70.6%)

### Temporal trends

**FIGURE 2:**
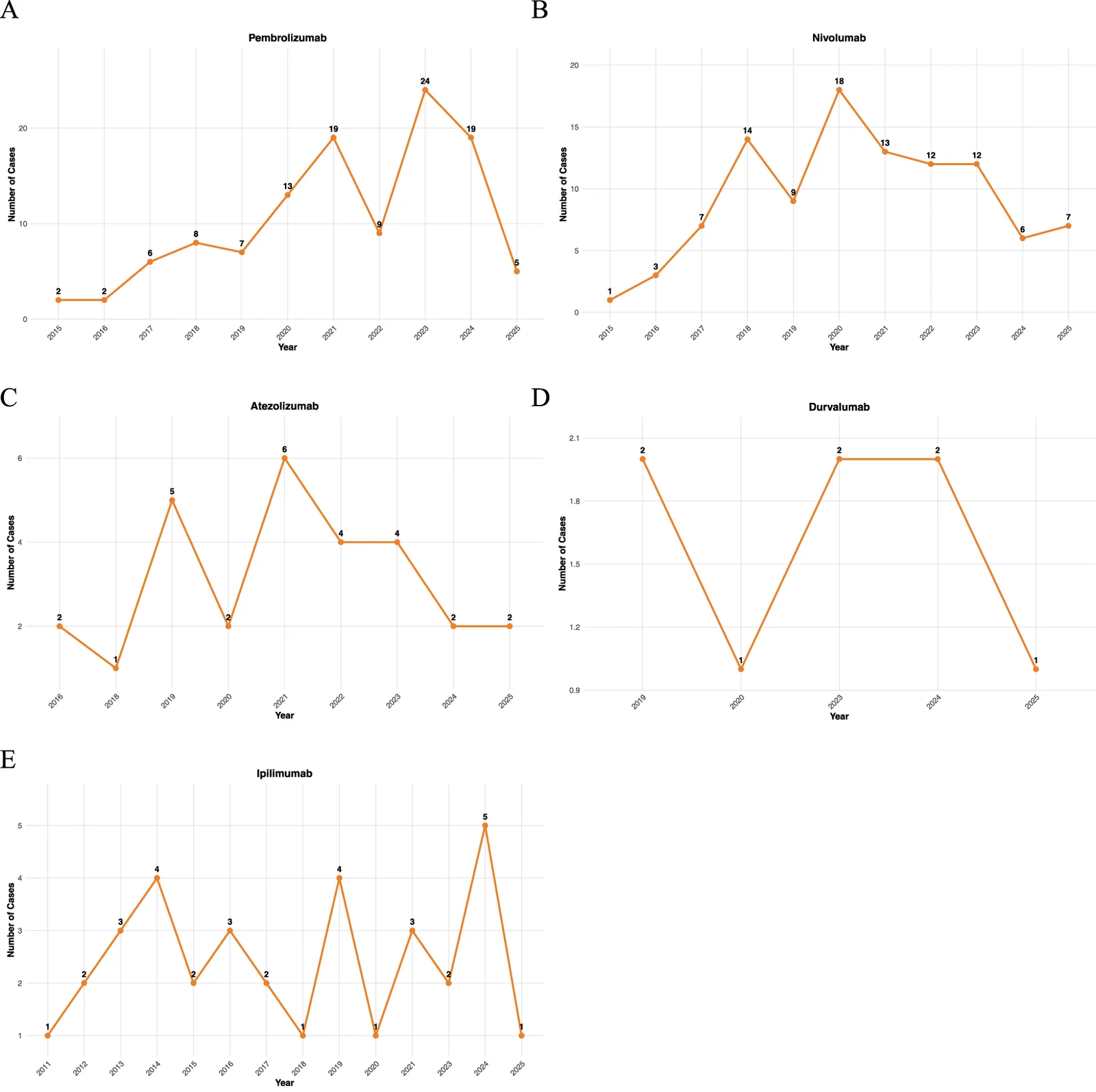
Temporal trends of ICI-related optic nerve adverse event reports **(A–E)** Show the annual temporal distribution of optic nerve-related adverse event reports associated with pembrolizumab **(A)**, nivolumab **(B)**, atezolizumab **(C)**, durvalumab **(D)**, and ipilimumab **(E)**. The number of reports increased over time for pembrolizumab and nivolumab, whereas fluctuating patterns were observed for atezolizumab, durvalumab, and ipilimumab.

A total of 53,522 pembrolizumab reports, 67,178 nivolumab reports, 23,394 atezolizumab reports, 13,246 durvalumab reports, and 18,488 ipilimumab reports were retrieved. Among these, 114, 102, 28, 8, and 34 optic nerve-related adverse event cases were identified, respectively (Supplementary Table S4–S8). As shown in Figure 2, pembrolizumab- and nivolumab-related reports increased over time, with peaks in 2023 and 2020, respectively. Atezolizumab-, durvalumab-, and ipilimumab-related reports showed fluctuating temporal patterns, with the highest numbers observed in 2021, 2023–2024, and 2024, respectively. Overall, the number of reports increased in recent years.

### Disproportionality analysis

**FIGURE 3:**
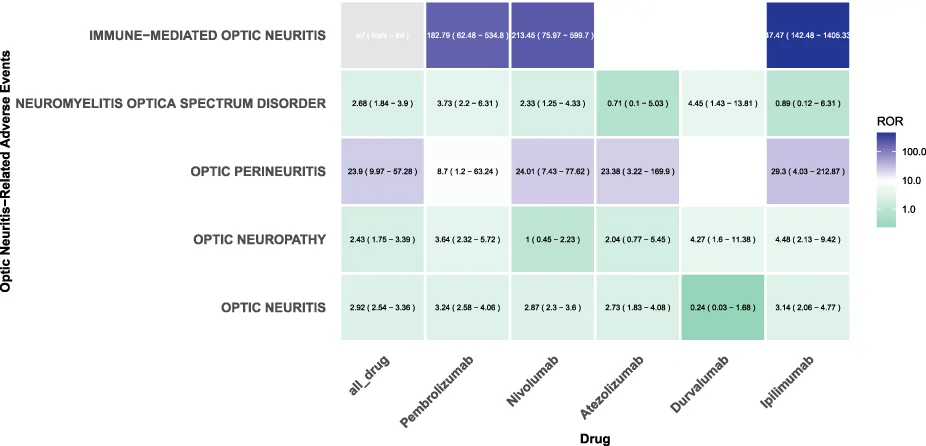
Signal detection for ICI-related optic nerve adverse events at the overall and PT levels. The figure presents a comprehensive analysis of safety signals. It shows the overall disproportionality analysis for optic nerve-related adverse events associated with immune checkpoint inhibitors, including ROR, PRR, and EBGM values. Concurrently, it displays the PT-level signal detection results for major optic nerve-related conditions, such as optic neuritis, optic neuropathy, optic perineuritis, and neuromyelitis optica spectrum disorder. Signal strength was assessed using standard pharmacovigilance metrics.

Disproportionality analysis revealed a significant signal for optic nerve-related adverse events associated with the five ICIs as a whole (ROR 3.02, 95% CI 2.69–3.40; PRR 3.02, χ^2^ = 371.35; EBGM 2.97, EBGM05 = 2.64) (Table 2). The specific case numbers for each PT were shown in Supplementary Table S9, and the overall signal results are presented in Figure 3. At the individual drug level, pembrolizumab, nivolumab, and ipilimumab generated positive signals, whereas atezolizumab showed an elevated but non-significant signal, and durvalumab did not reach the predefined threshold for a positive signal in the analysis of this dataset.

**TABLE 2:** Signal detection analysis of optic neuritis-related adverse reactions induced by immune checkpoint inhibitors.

Drug	Cases (n)	ROR (95%CI)	PRR (X2)	EBGM (EBGM05)
ICIs (all drug)	286	3.02 (2.69–3.4)	3.02 (371.35)	2.97 (2.64)
Pembrolizumab	114	3.53 (2.94–4.24)	3.53 (206.47)	3.5 (2.92)
Nivolumab	102	2.74 (2.25–3.33)	2.74 (111.63)	2.72 (2.24)
Atezolizumab	28	2.46 (1.72–3.52)	2.46 (25.83)	2.45 (1.71)
Durvalumab	8	1.37 (0.68–2.73)	1.37 (0.78)	1.37 (0.68)
Ipilimumab	34	3.59 (2.58–5.01)	3.59 (65.25)	3.58 (2.57)

We performed an extended search of all optic nerve-related terms based on the broad version of the MedDRA Standardised MedDRA Query (SMQ) for optic neuritis (ID: 20000023), including retrobulbar neuritis, papilledema, papillitis, neuroretinitis, ischaemic optic neuropathy, optic neuritis, optic neuropathy, optic perineuritis, immune-mediated optic neuritis, neuromyelitis optica spectrum disorder and so on. The search was restricted to reports in which the five target ICIs were coded as the primary suspect (PS) drug. Among the additionally searched terms, retrobulbar neuritis, papilledema, papillitis, and neuroretinitis yielded zero cases. Ischaemic optic neuropathy was excluded from the primary analysis due to its mechanistic irrelevance to the core toxicity pathways of ICIs and the presence of confounding factors in the sporadic reports identified. Ultimately, the PT-level analysis identified optic neuritis, optic neuropathy, optic perineuritis, immune-mediated optic neuritis, and neuromyelitis optica spectrum disorder as the main detected events. The PT-level signal results are presented in Figure 3. Among these, immune-mediated optic neuritis and optic perineuritis exhibited particularly strong signals across several ICIs.

### Time-to-onset and weibull analysis

**FIGURE 4:**
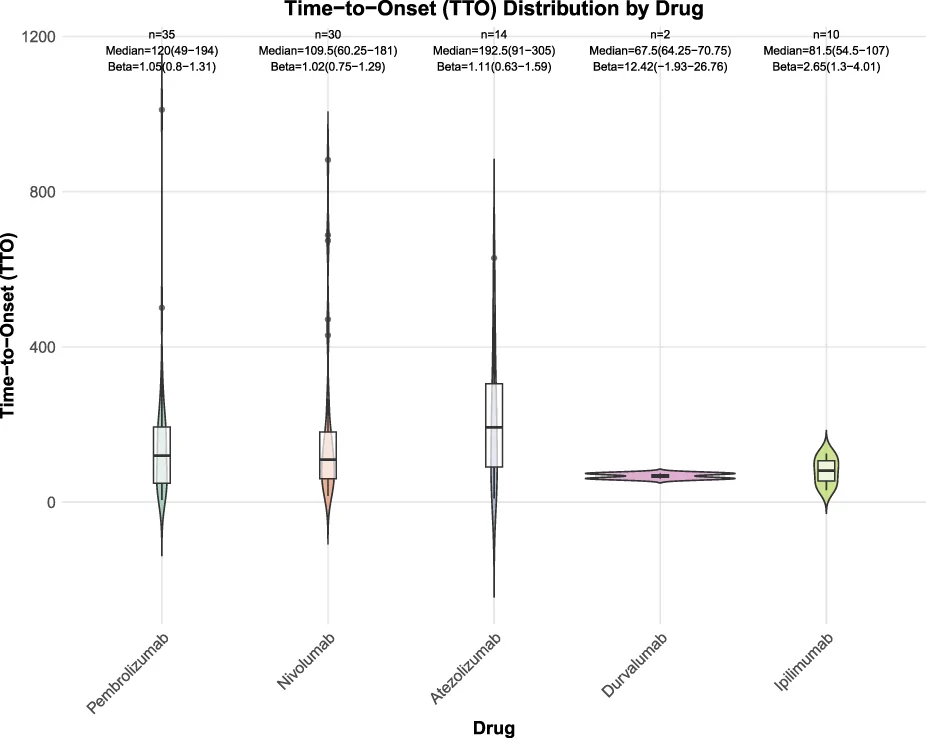
Time-to-onset analysis of ICI-related optic nerve adverse events. All five agents exhibited a wear-out failure pattern, indicating that the risk of onset increased over time. Specifically, the median TTO was 120 days with a shape parameter β of 1.05 for pembrolizumab; 109.5 days with β = 1.02 for nivolumab; the longest median TTO (192.5 days) with β = 1.11 for atezolizumab; a relatively short median TTO (81.5 days) and the highest β value (2.65) for ipilimumab; and a median TTO of 67.5 days with β = 12.42 for durvalumab.

Time-to-onset varied across drugs and age groups (Figure 4). The median time to onset was 120 days for pembrolizumab, 109.5 days for nivolumab, 192.5 days for atezolizumab, 67.5 days for durvalumab, and 81.5 days for ipilimumab. The shortest median time to onset was generally observed in the 65–85-year group for pembrolizumab, nivolumab, and ipilimumab, whereas atezolizumab and durvalumab showed shorter onset times in the 18–64.9-year group.

**FIGURE 5:**
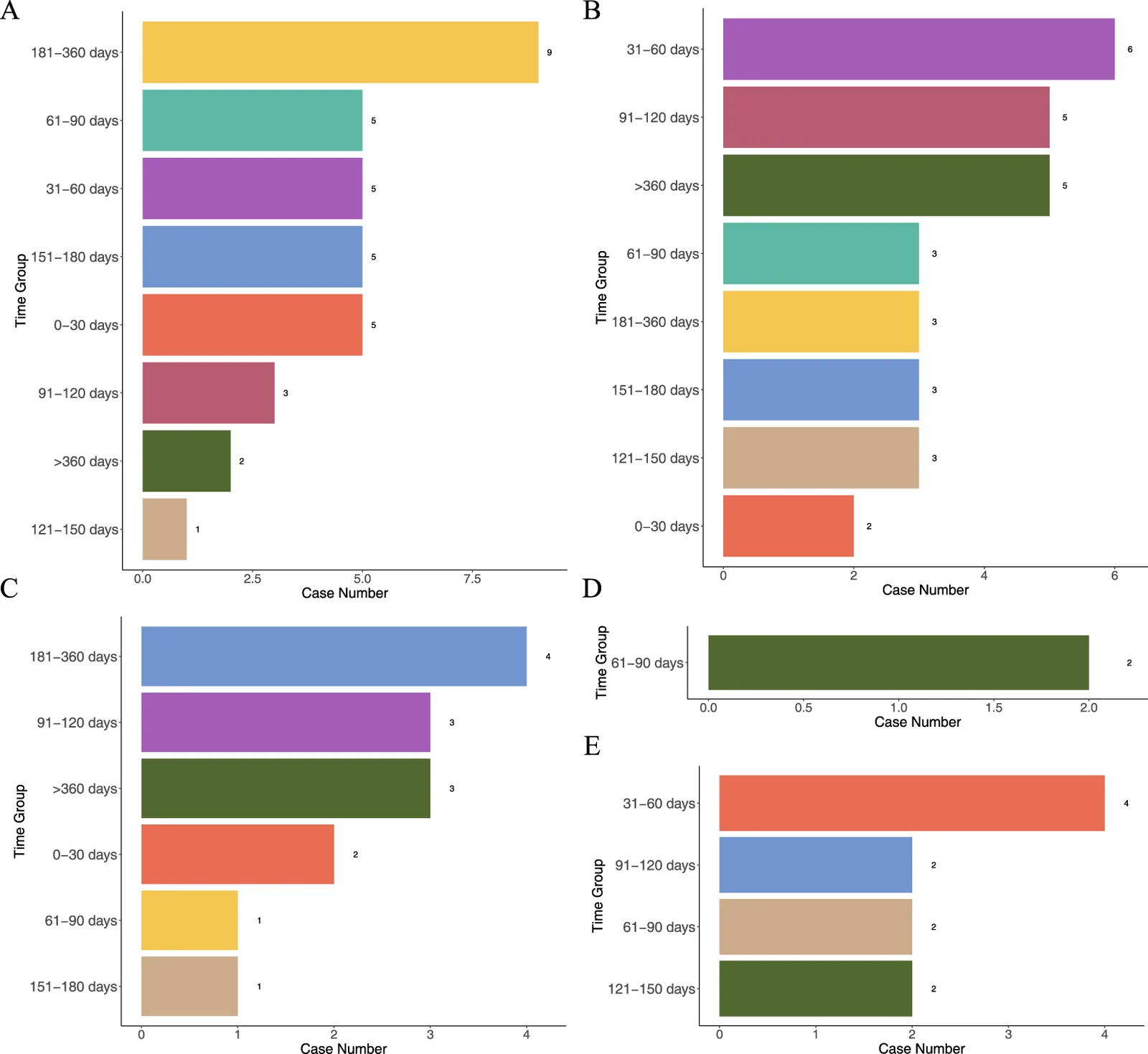
Weibull distribution analysis of ICI-related optic nerve adverse events. **(A–E)** illustrate the Weibull distribution analysis for optic nerve-related adverse events associated with pembrolizumab **(A)**, nivolumab **(B)**, atezolizumab **(C)**, durvalumab **(D)**, and ipilimumab **(E)**. Panels include the Weibull probability plots and parameter estimates used to characterize the hazard pattern over time. The results suggest a wear-out failure pattern, indicating an increasing risk of adverse events with prolonged exposure.

Weibull analysis showed that all five ICIs exhibited a “wear-out failure” time-to-onset pattern, suggesting that the reporting probability of optic nerve-related adverse events might increase gradually with prolonged exposure (Figure 5; Table 3).

**TABLE 3:** Weibull distribution fitting and failure patterns of time to onset for immune checkpoint inhibitor-induced optic neuritis.

Drugs	Available	Median (IQR)	Weibull distribution		Failure.Type
	Cases		Alpha [95%CI]	Beta [95%CI]	
Pembrolizumab	35	120 (49–194)	164.16 (109.53–218.8)	1.05 (0.8–1.31)	Wear-out failure
Nivolumab	30	109.5 (60.25–181)	195.37 (122.63–268.1)	1.02 (0.75–1.29)	Wear-out failure
Atezolizumab	14	192.5 (91–305)	229.99 (116.12–343.87)	1.11 (0.63–1.59)	Wear-out failure
Durvalumab	2	67.5 (64.25–70.75)	70.47 (62.17–78.78)	12.42 (-1.93–26.76)	Wear-out failure
Ipilimumab	10	81.5 (54.5–107)	89.65 (67.63–111.66)	2.65 (1.3–4.01)	Wear-out failure

### Clinical features and model importance analysis

**FIGURE 6:**
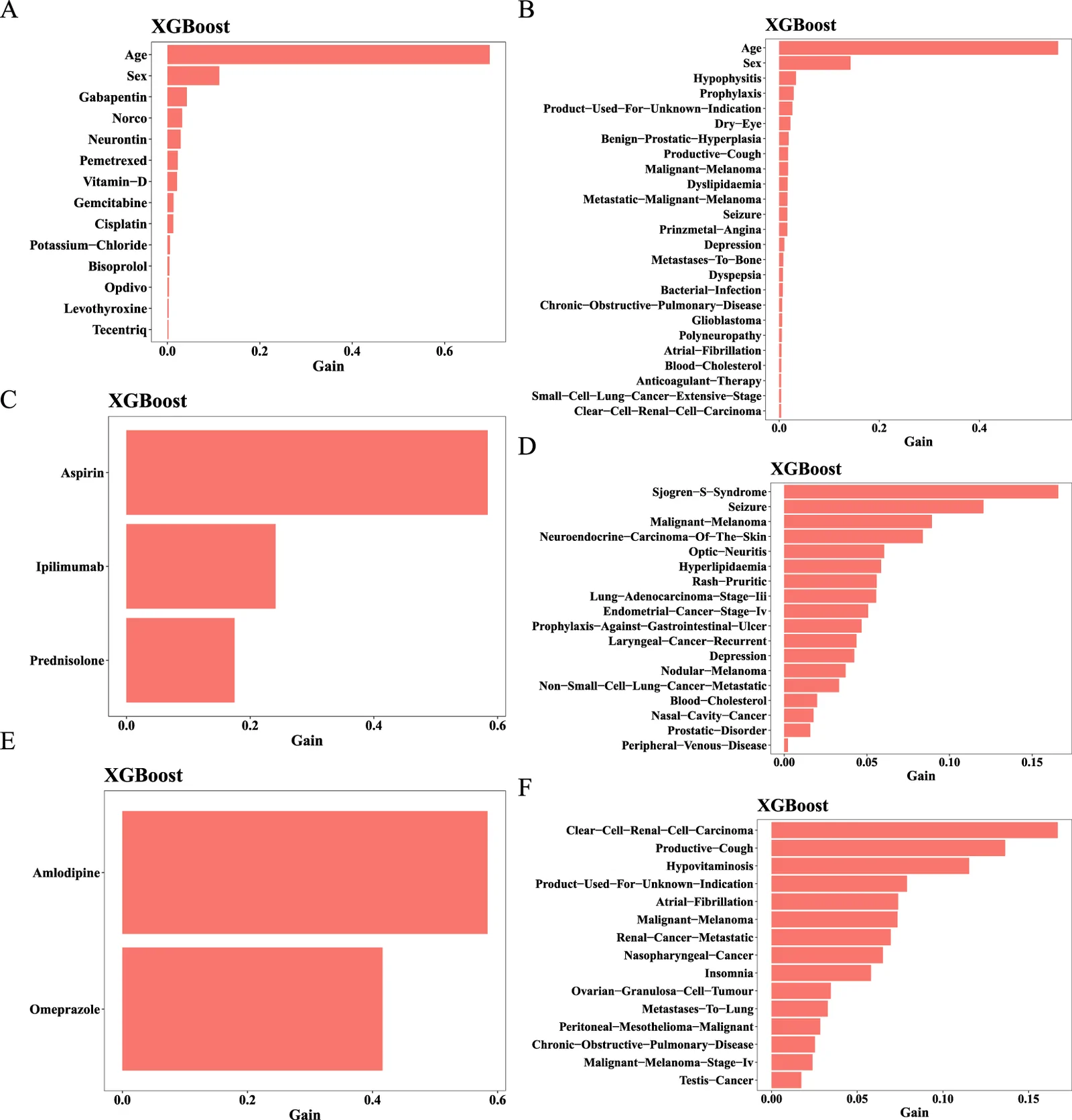
XGBoost feature importance analysis for ICI-related optic nerve adverse events **(A–F)** Present the XGBoost-derived feature importance ranking for features associated with optic nerve-related adverse events **(A,B)** Show the combined ICI analysis **(C,D)** show the pembrolizumab-specific model; and **(E,F)** show the nivolumab-specific model. Features are ranked according to their contribution to model prediction. This ranking reflects predictive contribution within the machine learning model.

Multivariate regression analysis showed that age, sex, selected concomitant medications (Table 4), and comorbidities (Table 5) were statistically associated with reports of optic nerve-related adverse events. The corresponding regression results are provided in the supplementary tables, and XGBoost analysis further ranked age as the most important feature, followed by sex and selected concomitant medications or comorbidities (Figures 6A,B).

**TABLE 4:** Influencing factors (concomitant medications) for optic neuritis-related adverse events induced by the five ICIs.

Characteristics	P Value	OR(95% CI)
Opdivo	<0.05	0.74 (0.56–0.97)
Cisplatin	<0.05	2.25 (1.43–3.36)
Tecentriq	<0.05	0.42 (0.17–0.86)
Pemetrexed	<0.05	1.94 (1.06–3.21)
Gabapentin	<0.05	3.59 (2.4–5.14)
Bisoprolol	<0.05	2.08 (1.16–3.39)
Potassium chloride	<0.05	1.91 (1.09–3.06)
Gemcitabine	<0.05	2.93 (1.51–5.04)
Levothyroxine	<0.05	0.39 (0.14–0.85)
Neurontin	<0.05	5.23 (2.93–8.54)
Norco	<0.05	6.39 (3.85–9.91)
Vitamin D	<0.05	9.23 (4.19–17.33)

**TABLE 5:** Influencing factors (comorbidities) for optic neuritis-related adverse events induced by the five ICIs.

Characteristics	P Value	OR(95% CI)
Malignant melanoma	<0.05	2.01 (1.57–2.54)
Product used for unknown indication	<0.05	0.72 (0.55–0.91)
Metastatic malignant melanoma	<0.05	2.08 (1.45–2.87)
Gastrointestinal carcinoma	<0.05	15.75 (0.89–70.53)
Clear cell renal cell carcinoma	<0.05	3.44 (1.56–6.46)
Metastases to kidney	<0.05	15.91 (0.9–71.25)
Small cell lung cancer extensive stage	<0.05	2.65 (1.45–4.41)
Prinzmetal angina	<0.05	115.62 (48.52–232.34)
Dyslipidaemia	<0.05	3.96 (1.8–7.45)
Lung adenocarcinoma recurrent	<0.05	12.07 (2–37.72)
Glioblastoma	<0.05	5.75 (2.05–12.48)
Hypovitaminosis	<0.05	16.14 (3.99–42.48)
Depression	<0.05	4.46 (2.43–7.43)
Seizure	<0.05	12.01 (5.13–23.5)
Prophylaxis against gastrointestinal ulcer	<0.05	3.77 (1.16–8.82)
Myasthenia gravis	<0.05	16.61 (2.74–52.04)
Hypothyroidism	<0.05	2.26 (1.2–3.83)
Dry eye	<0.05	14.69 (6.26–28.8)
Premedication	<0.05	2.51 (1.14–4.71)
Benign prostatic hyperplasia	<0.05	9.05 (4.48–16.15)
Chronic obstructive pulmonary disease	<0.05	3.72 (1.77–6.77)
Productive cough	<0.05	12.7 (5.01–26.03)
Dyspepsia	<0.05	4.53 (2.16–8.25)
Hypophysitis	<0.05	35.93 (15.26–70.82)
Atrial fibrillation	<0.05	3.57 (1.62–6.72)
Lung adenocarcinoma stage iii	<0.05	8.46 (2.61–19.85)
Rheumatoid arthritis	<0.05	5.67 (1.41–14.84)
Dizziness	<0.05	15.52 (4.78–36.56)
Prophylaxis	<0.05	0.05 (0–0.24)
Metastases to bone	<0.05	4.46 (2.3–7.71)
Anxiety	<0.05	2.38 (1.23–4.12)
Anticoagulant therapy	<0.05	5.04 (1.8–10.93)
Blood cholesterol	<0.05	17.08 (6.08–37.2)
Bacterial infection	<0.05	52.94 (18.72–116.64)
Polyneuropathy	<0.05	46.97 (16.62–103.28)
Rash pruritic	<0.05	26.64 (6.56–70.38)
Seasonal allergy	<0.05	5.97 (1.84–13.99)
Hypocalcaemia	<0.05	7.89 (2.44–18.5)
Hypomagnesaemia	<0.05	3.03 (0.94–7.11)
Hepatitis	<0.05	9.94 (1.65–31.03)

At the individual drug level, each drug exhibited distinct clinical association features. In the pembrolizumab-specific model (Figures 6C,D), aspirin and Sjögren’s syndrome ranked relatively high in feature importance (Supplementary Tables S10, S11). In the nivolumab-specific model (Figures 6E,F), amlodipine and clear cell renal cell carcinoma showed relatively high feature importance (Supplementary Tables S12, S13).

The XGBoost results for atezolizumab, durvalumab, and ipilimumab were presented in Supplementary Figure S1 due to figure size constraints. Specifically, the corresponding feature importance plots were shown in Supplementary Figure S1, and the complete model outputs were provided in Supplementary Table S14–S19. In the atezolizumab model, the top-ranked variables by feature importance were age, sex, and bone metastasis; in the durvalumab model, premedication and glioblastoma showed high predictive contribution; in the ipilimumab model, age, sex, pembrolizumab (all coded as concomitant or secondary suspect drugs), pyrexia, and malignant pleural mesothelioma showed relatively high feature importance.

## Discussion

ICIs are associated with a broad spectrum of ocular toxicities, including uveitis, dry eye disease, retinopathy, and neuro-ophthalmic complications (Wladis and Kambam, 2022). Among these events, optic nerve toxicity is an inflammatory optic neuropathy and remains one of the most important causes of visual loss (Žorić and Čolak, 2025). Although ICIs are increasingly recognized as potential triggers of optic nerve toxicity and related optic nerve disorders, systematic pharmacovigilance evidence on this issue remains limited. Using FAERS data from the first quarter of 2004 to the second quarter of 2025, the present study comprehensively evaluated optic nerve toxicity associated with five ICIs, with the aim of clarifying reporting characteristics, temporal patterns, signal strength, time-to-onset profiles, and associated factors. Our findings help fill an important evidence gap and may support risk awareness and safety monitoring in clinical practice.

Several important findings emerged from this study. First, a significant disproportionality signal was detected for optic nerve toxicity in the pooled analysis of the five ICIs, and positive signals were observed at the individual drug level for pembrolizumab, nivolumab, and ipilimumab. Preferred Term-level analyses further identified optic neuritis, optic neuropathy, optic perineuritis, and neuromyelitis optica spectrum disorder as the main reported events, with particularly strong signals for immune-mediated optic neuritis and optic perineuritis in some ICIs. These findings are broadly consistent with previous case reports and systematic reviews indicating that neuro-ophthalmic toxicity, although uncommon, represents a clinically meaningful immune-related adverse event associated with checkpoint blockade (Pietris et al., 2023; Yu et al., 2020). Case-based evidence has also suggested that atezolizumab may precipitate neuromyelitis optica spectrum disorder or related demyelinating optic nerve injury in susceptible patients supporting the biological plausibility of our observations (Pedrero Prieto et al., 2024).

Second, baseline characteristics analysis indicated that optic nerve toxicity was mainly reported in middle-aged and older adults, with the United States contributing the largest number of reports. Sex-related differences were also observed across drugs: female reports were relatively more frequent in the atezolizumab and ipilimumab groups, whereas male reports predominated in the pembrolizumab, nivolumab, and durvalumab groups. In addition, age ranked as the most important feature in the XGBoost models, followed by sex. These findings are noteworthy because prior studies have suggested that older age, male sex, and poorer visual function at presentation may be associated with less favorable outcomes in acute optic neuritis (Küchlin et al., 2025), whereas female sex may predict poorer prognosis in myelin oligodendrocyte glycoprotein antibody-associated disease, and advanced age may limit functional recovery in NMOSD-related optic neuritis and idiopathic optic neuritis (Min et al., 2024). Population-based evidence has further shown racial, ethnic, and sex-related disparities in optic neuritis epidemiology, with substantial heterogeneity across subgroups (Abbass et al., 2025). Taken together, our results suggest that susceptibility and reporting patterns of ICI-associated optic nerve toxicity may differ by drug and patient subgroup. From a clinical perspective, closer ophthalmic surveillance may be warranted for women receiving atezolizumab or ipilimumab and for men receiving pembrolizumab, nivolumab, or durvalumab, although these findings should be interpreted cautiously and require external validation.

Third, the time-to-onset and Weibull analyses suggest that optic nerve toxicity associated with all five ICIs follows a wear-out failure pattern, indicating that the reporting risk increases over time (Asano et al., 2023). This pattern has practical implications. As exposure to ICIs is prolonged, delayed immune-mediated toxicities may become more apparent, particularly in patients who remain on treatment or receive maintenance therapy (Fletcher and Johnson, 2024; Zammit and Seront, 2024). Our temporal trend analysis also showed that the number of reports has increased in recent years. This likely reflects several concurrent factors: the expanding indications and use of ICIs in oncology, a growing population exposed to these agents, and improved awareness and reporting of ocular and neuro-ophthalmic immune-related adverse event (Pietris et al., 2023; Yu et al., 2020)s. Importantly, the increasing number of reports may also indicate that delayed or chronic ocular toxicities are becoming more clinically visible as survival improves and treatment duration lengthens (Fletcher and Johnson, 2024). These findings underscore the need for continued ophthalmic monitoring throughout the treatment course, rather than restricting surveillance to the early treatment phase.

Another notable finding concerns the associated factors identified in the combined models. Beyond age and sex, gabapentin, Norco, and linagliptin showed relatively high importance among concomitant medications, whereas erosive gastritis and hypophysitis ranked highly among comorbidities. These variables should not be interpreted as confirmed causal determinants; rather, they may represent clinically relevant markers of vulnerability, comorbidity burden, treatment complexity, or shared inflammatory pathways. Previous studies have shown that patients receiving combination therapies or experiencing severe immune-related adverse events may be more likely to develop serious neurological toxicities and have worse long-term outcomes (Mancone et al., 2018). The signal for Norco is of particular interest because it contains acetaminophen, and recent work has raised concerns about a potential association between acetaminophen exposure and poorer long-term outcomes in patients treated with ICIs, although the mechanism remains uncertain (de Lemos et al., 2025).

Among comorbidities, the prominence of hypophysitis is biologically plausible. Hypophysitis is a well-recognized endocrine immune-related adverse event of ICIs and may indicate a broader state of immune dysregulation (Patel et al., 2019). In severe cases, pituitary inflammation and associated sellar involvement may produce visual symptoms or visual field defects, while concomitant systemic immune activation may increase the likelihood of inflammatory injury affecting the optic nerve. Likewise, erosive gastritis may reflect an underlying inflammatory or stress-related systemic condition (Yang et al., 2024). Chronic gastrointestinal mucosal inflammation could theoretically amplify systemic immune activation and thereby increase susceptibility to extraintestinal immune-related toxicities. Although these interpretations remain speculative, they are consistent with the concept that concomitant medications and comorbidities may shape the occurrence and severity of ICI-related optic nerve toxicity through overlapping inflammatory, immune-modulatory, or organ-specific mechanisms. Therefore, the high-importance variables identified in this study may have potential value in clinical risk stratification and early monitoring.

However, it must be emphasized that the high-importance features identified by the XGBoost model should not be directly interpreted as causal risk factors. Taking nivolumab as an example, amlodipine and clear cell renal cell carcinoma ranked highly in the model. Given that nivolumab is the standard of care for advanced renal cell carcinoma (Choueiri et al., 2024) and that the prevalence of hypertension is extremely high in this patient population, the prominence of amlodipine likely reflects the management of this underlying comorbidity (Wang et al., 2023) rather than any intrinsic optic neurotoxicity. This illustrates the differential association profiles across different ICIs, which may require individualized interpretation and adjustment based on specific drug and patient characteristics. Furthermore, these findings suggest that future research should validate such associations in larger, prospective datasets and employ sensitivity analyses that exclude clear comorbidity proxy medications to further distinguish true causal factors from surrogate markers, thereby providing a more reliable foundation for clinical risk stratification.

Drug-specific analyses also suggested distinct profiles across ICIs. For pembrolizumab, aspirin and Sjögren’s syndrome were among the more influential features, whereas amlodipine and clear cell renal cell carcinoma ranked highly in the nivolumab model. In the supplementary analyses, age, sex, and bone metastasis were important contributors for atezolizumab; preoperative medication and glioblastoma were influential in the durvalumab model; and age, sex, pembrolizumab exposure, fever, and malignant pleural mesothelioma were among the leading features in the ipilimumab model. These differences may reflect heterogeneity in patient selection, cancer types, co-medication patterns, treatment sequencing, and underlying immune susceptibility. They also reinforce the view that ICI-associated optic nerve toxicity is unlikely to be explained by a single mechanism and instead may arise from multiple interacting host-, disease-, and treatment-related factors.

Furthermore, two notable findings from the drug-specific analyses further underscore the importance of confounding factors in signal interpretation. First, durvalumab did not generate a statistically significant disproportionality signal in this study. However, this result should not be directly equated with a lower risk of optic neurotoxicity. Durvalumab is currently approved primarily for consolidation therapy in unresectable stage III non-small cell lung cancer (NSCLC), perioperative treatment of resectable NSCLC, and limited-stage small cell lung cancer (Botticella et al., 2019; Mooradian et al., 2024). These indications typically correspond to patient populations with relatively earlier tumor stages and better performance status (Koczywas et al., 2013). In contrast, pembrolizumab, nivolumab, and ipilimumab are more widely used in advanced or metastatic solid tumors (Kaneko et al., 2024), where patients generally present with higher tumor burden, longer treatment duration, and more complex polypharmacy regimens (He et al., 2022). These fundamental differences in clinical utilization may contribute to distinct baseline characteristics and immune-related adverse event profiles across different ICI-exposed populations. Moreover, the extremely limited number of optic neurotoxicity events reported for durvalumab in FAERS may have further reduced the statistical power for signal detection. Therefore, the negative signal observed for durvalumab in this study more likely reflects the low incidence of adverse events and insufficient statistical power within its specific clinical context, rather than an inherently superior ocular safety profile.

Second, the emergence of prior pembrolizumab exposure as a highly ranked feature in the ipilimumab-specific model may reflect real-world sequential treatment trajectories. In the identified optic neurotoxicity cases, ipilimumab was consistently coded as the primary suspect drug, whereas pembrolizumab was recorded as a concomitant or secondary suspect medication. In clinical practice, it is a common therapeutic strategy for patients with advanced melanoma to receive pembrolizumab followed by ipilimumab-containing salvage regimens upon disease progression (Zimmer et al., 2017). However, FAERS lacks comprehensive treatment timeline data, making it difficult to determine whether the adverse event should be attributed to the current therapy, a delayed effect of prior treatment, or a cumulative effect of sequential exposures. Accordingly, this feature is more likely indicative of the complexity of patients’ treatment history rather than a direct exacerbation of ipilimumab toxicity by pembrolizumab. Collectively, these observations suggest that when comparing the safety profiles of different ICIs using real-world databases such as FAERS, it is essential to account for key confounders including the specific clinical indications, characteristics of the exposed populations, and prior treatment history. Future studies should consider incorporating treatment line and prior medication history into the analytical framework.

The potential clinical implications of our findings deserve emphasis. Although optic nerve toxicity is rare, it may lead to severe and sometimes irreversible visual impairment. Early recognition is therefore critical. Patients receiving ICIs, especially those with prolonged exposure or with clinical features of high importance identified in this study, may benefit from baseline ophthalmic assessment, prompt evaluation of new visual symptoms, and multidisciplinary management involving oncologists, ophthalmologists, and neurologists when necessary (Phuljhele et al., 2021; De Lott et al., 2022; Cuzzubbo et al., 2026). For patients with clinical features of high importance identified in this study, more proactive monitoring of visual acuity, color vision, pupillary responses, visual fields, and optic nerve function may be justified. Because optic nerve toxicity may also occur as part of broader demyelinating or autoimmune syndromes, clinicians should maintain vigilance for associated neurological manifestations.

This study has several limitations. First, as a spontaneous reporting database, FAERS is inherently subject to underreporting, overreporting, duplicate reporting, missing data, and variable data quality, all of which may introduce reporting bias. Second, this study did not include non-immune-mediated optic nerve events covered by the broad version of the SMQ (e.g., ischemic or infectious optic neuropathy). Although our search yielded no hits for these terms among PS reports of the five ICIs, the possibility remains that a very small number of cases were not captured due to the absence of corresponding PT codes. Furthermore, the inclusion of the Preferred Term “immune-mediated optic neuritis” may introduce circular reasoning bias, potentially inflating the estimated signal strength to some extent. Future studies should consider conducting sensitivity analyses using validated SMQs to evaluate the impact of different term selection strategies on the robustness of the results. Third, disproportionality analyses identify statistical reporting associations rather than causality, and the observed signals should not be interpreted as definitive evidence of a causal relationship. Fourth, detailed clinical information is limited in FAERS, including imaging findings, antibody status, ophthalmic examination results, treatment response, rechallenge outcomes, and severity grading, which constrains mechanistic and prognostic interpretation. Fifth, confounding by indication, cancer type, disease severity, concomitant therapies, and coexisting autoimmune conditions cannot be fully addressed. Sixth, the machine learning feature importance results indicate predictive contribution within the model rather than causal effect size, and these findings require external validation in independent datasets. Finally, because the study was based on real-world pharmacovigilance data, incidence rates could not be estimated directly.

Despite these limitations, this study provides a large-scale and systematic pharmacovigilance assessment of optic nerve toxicity associated with ICIs. In summary, pooled ICI exposure was associated with a significant signal for optic nerve toxicity, with positive signals particularly observed for pembrolizumab, nivolumab, and ipilimumab. The risk appeared to increase over time, and age, sex, selected concomitant medications, and comorbidities were identified as potentially important associated factors. These findings may help improve awareness of this rare but potentially vision-threatening toxicity and support earlier detection and risk-adapted monitoring. Future studies based on prospective cohorts, multicenter registries, and mechanistic investigations are needed to validate these signals, clarify causality, and refine prevention and management strategies for ICI-associated optic nerve toxicity.

## Data Availability

The datasets presented in this study can be found in online repositories. The names of the repository/repositories and accession number(s) can be found in the article/Supplementary Material.

## Supplementary material

SUPPLEMENTARY FIGURE S1 XGBoost feature importance analysis of optic nerve adverse events. **(A,B)** Show the XGBoost-derived feature importance ranking for the atezolizumab-specific model. **(C,D)** Show the ranking for the durvalumab-specific model. **(E,F)** Show the ranking for the ipilimumab-specific model. Features are ordered according to their relative contribution to prediction of optic nerve-related adverse events. This ordering reflects predictive contribution within the machine learning model.
