# Supplementary material for: Pharmacovigilance analysis of immune checkpoint inhibitor-associated optic nerve toxicity based on the FAERS database
Source: Front Pharmacol. 2026 Aug 24;17:1845963. doi: 10.3389/fphar.2026.1845963 (PMC13547466; doi:10.3389/fphar.2026.1845963)
Supplement: Supplementary file 1 [file Table1.docx]

Supplementary Table 1 ROR、PRR、BCPNN、BCPNN computational formula

| Method | Formula | Symbol Description | Standard |
| --- | --- | --- | --- |
| ROR | ROR = （a/b）/（c/d） | a: reports of target drug–target AE;b: reports of target drug–non-target AE;c: reports of other drugs–target AE;d: reports of other drugs–non-target AE | ROR > 1 and lower limit of 95% CI > 1 |
| PRR | PRR = [a/(a+b)]/[c/(c+d)]  χ2 = (ad-bc)2(a+b+c+d)/(a+b)(c+d)(a+c)(b+d) | a, b, c, d as above | a ≥ 3, PRR ≥ 2, χ² ≥ 4 |
| MGPS | EBGM = a + α / E + α + β E=[(a+c)·(a+b)]/(a+b+c+d) | a, b, c, d as above; E: expected count of target AE under independence assumption | EBGM05 > 2 and a > 0 |
| BCPNN | IC = log_2_ a·(a+b+c+d)/[(a+c)·(a+b)] | a, b, c, d as above | IC025 > 0 |
