# Supplementary material for: Pharmacovigilance analysis of immune checkpoint inhibitor-associated optic nerve toxicity based on the FAERS database
Source: Front Pharmacol. 2026 Aug 24;17:1845963. doi: 10.3389/fphar.2026.1845963 (PMC13547466; doi:10.3389/fphar.2026.1845963)
Supplement: Supplementary file 2 [file Table2.docx]

Supplementary Table 2 The 2 × 2 contingency table of the imbalance method

|  | Drug-related ADEs | Non-drug-related ADEs | total |
| --- | --- | --- | --- |
| drug | a | b | a + b |
| Non-drug | c | d | c + d |
| total | a + c | b + d | a + b + c + d |
