# Supplementary material for: Pharmacovigilance analysis of immune checkpoint inhibitor-associated optic nerve toxicity based on the FAERS database
Source: Front Pharmacol. 2026 Aug 24;17:1845963. doi: 10.3389/fphar.2026.1845963 (PMC13547466; doi:10.3389/fphar.2026.1845963)
Supplement: Supplementary file 3 [file Table3.docx]

Supplementary Table 3. Variables included in the XGBoost models for each ICI analysis

| Analysis | Initial Variable Pool |
| --- | --- |
| Alldrug-drug | AGE, SEX, OPDIVO, YERVOY, CISPLATIN, TECENTRIQ, PEMETREXED, GABAPENTIN, BISOPROLOL, POTASSIUM.CHLORIDE, AMLODIPINE, GEMCITABINE, LEVOTHYROXINE, NEURONTIN, NORCO, VITAMIN.D |
| Alldrug-disease | AGE, SEX, MALIGNANT.MELANOMA, PRODUCT.USED.FOR.UNKNOWN.INDICATION, LUNG.NEOPLASM.MALIGNANT, TESTIS.CANCER, RENAL.CANCER, METASTATIC.MALIGNANT.MELANOMA, RENAL.CELL.CARCINOMA, OESOPHAGEAL.CARCINOMA, GASTROINTESTINAL.CARCINOMA, METASTATIC.RENAL.CELL.CARCINOMA, CLEAR.CELL.RENAL.CELL.CARCINOMA, METASTASES.TO.KIDNEY, SMALL.CELL.LUNG.CANCER.EXTENSIVE.STAGE, PRINZMETAL.ANGINA, DYSLIPIDAEMIA, GASTRITIS.EROSIVE, LUNG.ADENOCARCINOMA.RECURRENT, SJOGREN.S.SYNDROME, GLIOBLASTOMA, HYPOVITAMINOSIS, DEPRESSION, SEIZURE, PROPHYLAXIS.AGAINST.GASTROINTESTINAL.ULCER, NEUROENDOCRINE.CARCINOMA.OF.THE.SKIN, PERIPHERAL.VENOUS.DISEASE, MYASTHENIA.GRAVIS, OPTIC.NEURITIS, HYPOTHYROIDISM, DRY.EYE, PREMEDICATION, MALIGNANT.MELANOMA.STAGE.IV, BENIGN.PROSTATIC.HYPERPLASIA, CHRONIC.OBSTRUCTIVE.PULMONARY.DISEASE, PRODUCTIVE.COUGH, SQUAMOUS.CELL.CARCINOMA.OF.THE.ORAL.CAVITY, DYSPEPSIA, CUTANEOUS.T.CELL.LYMPHOMA, ATYPICAL.MYCOBACTERIAL.INFECTION, OVARIAN.GRANULOSA.CELL.TUMOUR, HYPOPHYSITIS, ATRIAL.FIBRILLATION, NODULAR.MELANOMA, NASAL.CAVITY.CANCER, ALVEOLAR.SOFT.PART.SARCOMA.METASTATIC, LUNG.ADENOCARCINOMA.STAGE.III, LUNG.SQUAMOUS.CELL.CARCINOMA.STAGE.III, RHEUMATOID.ARTHRITIS, DIZZINESS, PROPHYLAXIS, METASTASES.TO.BONE, PROSTATIC.DISORDER, ANXIETY, ANTICOAGULANT.THERAPY, BLOOD.CHOLESTEROL, LOCALISED.INFECTION, BACTERIAL.INFECTION, POLYNEUROPATHY, RASH.PRURITIC, SEASONAL.ALLERGY, ORAL.HERPES, HYPOCALCAEMIA, HYPOMAGNESAEMIA, HER2.POSITIVE.BREAST.CANCER, HEPATITIS, PULMONARY.OEDEMA, TONSILLITIS, PERITONSILLAR.ABSCESS, LARYNGEAL.CANCER.RECURRENT |
| Pembrolizumab-drug | IPILIMUMAB, ASPIRIN, PREDNISOLONE |
| Pembrolizumab-disease | MALIGNANT.MELANOMA, ADENOCARCINOMA.OF.COLON, PROSTATE.CANCER, LUNG.ADENOCARCINOMA.RECURRENT, SJOGREN.S.SYNDROME, COLON.CANCER, RHINITIS.ALLERGIC, DEPRESSION, SEIZURE, PROPHYLAXIS.AGAINST.GASTROINTESTINAL.ULCER, NEUROENDOCRINE.CARCINOMA.OF.THE.SKIN, CORONARY.ARTERY.DISEASE, PERIPHERAL.VENOUS.DISEASE, MYASTHENIA.GRAVIS, CEREBRAL.INFARCTION, TUMOUR.PAIN, OPTIC.NEURITIS, ENDOMETRIAL.CANCER.STAGE.IV, CUTANEOUS.T.CELL.LYMPHOMA, HYPERLIPIDAEMIA, NODULAR.MELANOMA, NASAL.CAVITY.CANCER, NON.SMALL.CELL.LUNG.CANCER.METASTATIC, LUNG.ADENOCARCINOMA.STAGE.III, LUNG.SQUAMOUS.CELL.CARCINOMA.STAGE.III, RHEUMATOID.ARTHRITIS, METASTATIC.GASTRIC.CANCER, PROSTATIC.DISORDER, ANTICOAGULANT.THERAPY, BLOOD.CHOLESTEROL, ADENOCARCINOMA, RASH.PRURITIC, VULVAL.CANCER, LARYNGEAL.CANCER.RECURRENT |
| Nivolumab-drug | AMLODIPINE, OMEPRAZOLE |
| Nivolumab-disease | MALIGNANT.MELANOMA, PRODUCT.USED.FOR.UNKNOWN.INDICATION, TESTIS.CANCER, GASTROINTESTINAL.CARCINOMA, METASTASES.TO.KIDNEY, CLEAR.CELL.RENAL.CELL.CARCINOMA, LUNG.SQUAMOUS.CELL.CARCINOMA.STAGE.IV, METASTASES.TO.LUNG, HYPOVITAMINOSIS, UVEAL.MELANOMA, RENAL.CANCER.METASTATIC, BENIGN.PROSTATIC.HYPERPLASIA, CHRONIC.OBSTRUCTIVE.PULMONARY.DISEASE, PRODUCTIVE.COUGH, GLIOBLASTOMA, OVARIAN.GRANULOSA.CELL.TUMOUR, ATRIAL.FIBRILLATION, MALIGNANT.MELANOMA.STAGE.IV, NEOPLASM.MALIGNANT, ADRENAL.INSUFFICIENCY, INSOMNIA, PERITONEAL.MESOTHELIOMA.MALIGNANT, DYSLIPIDAEMIA, PRURITUS, NASOPHARYNGEAL.CANCER |
| Ipilimumab-drug | AGE, SEX, PEMBROLIZUMAB |
| Ipilimumab-disease | AGE, SEX, MALIGNANT.MELANOMA, PLEURAL.MESOTHELIOMA.MALIGNANT, PYREXIA, CEREBROVASCULAR.ACCIDENT.PROPHYLAXIS, DIZZINESS, MALIGNANT.MELANOMA.STAGE.IV, HYPOPHYSITIS |
| Durvalumab-disease | GLIOBLASTOMA, PREMEDICATION |
| Atezolizumab-drug | AGE, SEX, ATEZOLIZUMAB, TECENTRIQ |
| Atezolizumab-disease | AGE, SEX, SMALL.CELL.LUNG.CANCER.EXTENSIVE.STAGE, DYSLIPIDAEMIA, GASTRITIS.EROSIVE, SQUAMOUS.CELL.CARCINOMA.OF.THE.ORAL.CAVITY, ATYPICAL.MYCOBACTERIAL.INFECTION, ALVEOLAR.SOFT.PART.SARCOMA.METASTATIC, SEASONAL.ALLERGY, DEPRESSION, HEADACHE, ORAL.HERPES, ANXIETY, ARTHRALGIA, HYPOCALCAEMIA, RASH, HYPOMAGNESAEMIA, HER2.POSITIVE.BREAST.CANCER, HEPATITIS, PULMONARY.OEDEMA, FEBRILE.NEUTROPENIA, TONSILLITIS, PERITONSILLAR.ABSCESS, ABDOMINAL.DISCOMFORT, ARTHRITIS, METASTASES.TO.BONE |
